# Electrochemical Li Topotactic Reaction in Layered SnP_3_ for Superior Li-Ion Batteries

**DOI:** 10.1038/srep35980

**Published:** 2016-10-24

**Authors:** Jae-Wan Park, Cheol-Min Park

**Affiliations:** 1 School of Materials Science and Engineering, Kumoh National Institute of Technology, 61 Daehak-ro, Gumi, Gyeongbuk 39177, Republic of Korea

## Abstract

The development of new anode materials having high electrochemical performances and interesting reaction mechanisms is highly required to satisfy the need for long-lasting mobile electronic devices and electric vehicles. Here, we report a layer crystalline structured SnP_3_ and its unique electrochemical behaviors with Li. The SnP_3_ was simply synthesized through modification of Sn crystallography by combination with P and its potential as an anode material for LIBs was investigated. During Li insertion reaction, the SnP_3_ anode showed an interesting two-step electrochemical reaction mechanism comprised of a topotactic transition (0.7–2.0 V) and a conversion (0.0–2.0 V) reaction. When the SnP_3_-based composite electrode was tested within the topotactic reaction region (0.7–2.0 V) between SnP_3_ and Li_*x*_SnP_3_ (*x* ≤ 4), it showed excellent electrochemical properties, such as a high volumetric capacity (1st discharge/charge capacity was 840/663 mA h cm^−3^) with a high initial coulombic efficiency, stable cycle behavior (636 mA h cm^−3^ over 100 cycles), and fast rate capability (550 mA h cm^−3^ at 3C). This layered SnP_3_ anode will be applicable to a new anode material for rechargeable LIBs.

Li-ion batteries (LIBs) are used in high-end consumer electronic products, and are one of the representative energy sources for electric vehicles(EV), hybrid EV, and portable electronic devices. Graphite has been widely used as an anode material for LIBs, but it has a small theoretical capacity (372 mA h g^−1^ or ca. 840 mA h cm^−3^)^1^,^2^,^3^,^4^. Therefore, Sn-based materials have been suggested as representative alternative anode materials for LIBs because Sn can react reversibly with large amounts of Li, forming Li_4.25_Sn at room temperature, although Sn anodes show poor cycling behavior resulting from the huge volume distortions (~300%) during Li insertion/extraction^1^,^2^,^5^,^6^,^7^,^8^. To circumvent these detrimental effects, Sn-M alloys and Sn-based oxides have been suggested as alternative materials^9^,^10^,^11^,^12^,^13^,^14^,^15^,^16^,^17^,^18^,^19^,^20^,^21^,^22^,^23^,^24^,^25^,^26^,^27^. Although the aforementioned materials show enhanced cycling behavior, they can also exhibit small reversible capacities caused by use of inactive metals, or high initial capacity losses due to the formation of an inactive matrix phase (Li_2_O) during the first lithiation.

Recently, P-based materials have also been suggested as alternative anodes for LIBs, because of their interesting 2D-layer or 3D-channel structures^28^,^29^,^30^,^31^,^32^,^33^. These intriguing crystalline structures enable Li to diffuse easily within them. In addition, Li can react reversibly with P, forming Li_3_P phase, due to the high Li-ion conductivity and interesting puckered layer structure of P, in contrast with the inactive Li_2_O formed in Sn-based oxides^29^,^30^,^31^,^32^. In previous studies, a quasi-topotactic Li intercalation mechanism in a MnP_4_ electrode was demonstrated by Souza *et al.*^30^. Additionally, our group reported electrochemical Li quasi-intercalation in puckered layer structured black P that was synthesized by a simple mechanochemical process, which served as a stepping stone for recent numerous black P-based researches^31^. Furthermore, Li topotactic reactions in 3D-framework structured SiP_2_ and 3D-channel structured VP have also been reported^32^,^33^. However, although these materials showed stable cycling performances, they had relatively low reversible capacities. Therefore, new metal phosphide-based materials having interesting reaction mechanisms and high electrochemical performances are required.

In this study, we elected to examine SnP_3_ because it has an interesting layered crystalline structure, which means that interesting Li insertion/extraction behavior was expected. SnP_3_ was simply synthesized by a high energy ball milling (HEBM) process, producing a material whose electrochemical properties for LIBs were investigated. Furthermore, the electrochemical Li reaction in SnP_3_ was thoroughly investigated using various analytical techniques, including *ex situ* X-ray diffraction (XRD), X-ray absorption near edge structure (XANES), and extended X-ray absorption fine structure (EXAFS) analyses at the Sn K-edge, along with a differential capacity plot (DCP). On the basis of the reaction mechanism of the SnP_3_ electrode, we propose its utilization as a high performance LIB anode material.

## Results and Discussions

**Figure 1:**
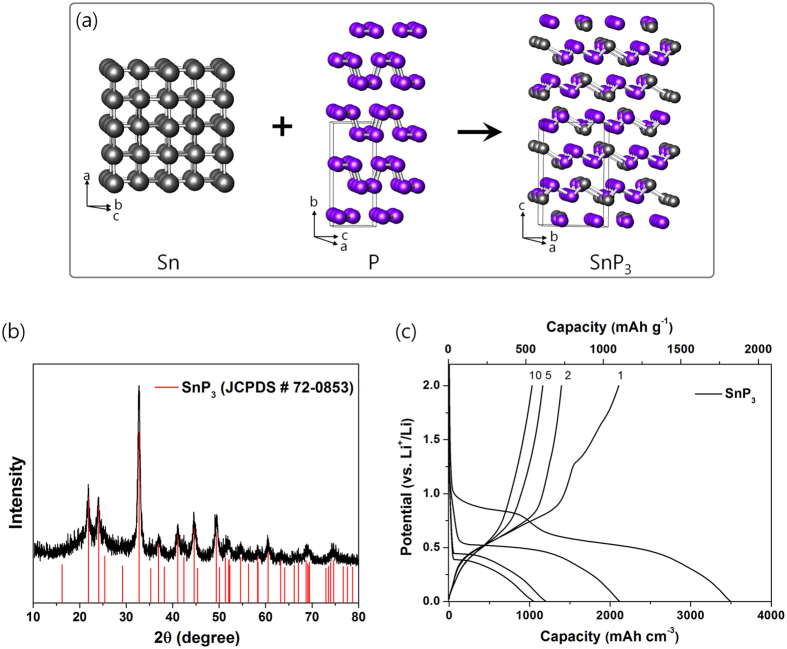
Synthesis and electrochemical performance of layer-structured SnP_3_. (**a**) Crystalline structure of the layer-structured SnP_3_ combining tetragonal Sn with orthorhombic P. (**b**) XRD pattern of synthesized SnP_3_. (**c**) Electrochemical voltage profile of layer-structured SnP_3_ electrode at a current rate of 100 mA g^−1^ (voltage range: 0.0–2.0 V).

SnP_3_ has an interesting layered rhombohedral crystalline structure (R-3m, a = 7.378 Å, c = 10.512 Å), which was formed by combining tetragonal Sn with puckered layer-structured P as shown in Fig. 1a^34^. The interesting layered structure of SnP_3_ enables facile Li diffusion and storage within its structure. Figure 1b shows the XRD pattern of the synthesized SnP_3_, the peaks of which agreed with those of the SnP_3_ standard (JCPDS # 72-0853) with no impurities observed. The voltage profile of the SnP_3_ electrode is shown in Fig. 1c. The SnP_3_ electrode displayed high discharge/charge capacities of 1820/1097 mA h g^−1^ (3495/2106 mA h cm^−3^), with an initial coulombic efficiency of 60.3%. Despite these high capacities, the capacity retention after the 10th cycle was ca. 48.7% of the first charge capacity. Sn and P (black) electrodes also showed high initial discharge and charge capacities (Fig. S1a,b in Supporting Information). However, they demonstrated extremely poor capacity retentions of 14.7% and 3.9% after the 10th cycle, respectively. The very poor capacity retentions of the Sn and P electrodes were caused by the huge volume distortion by the formation of the Li_4.25_Sn (~300%) or Li_3_P (~315%) alloying phases, respectively.

**Figure 2:**
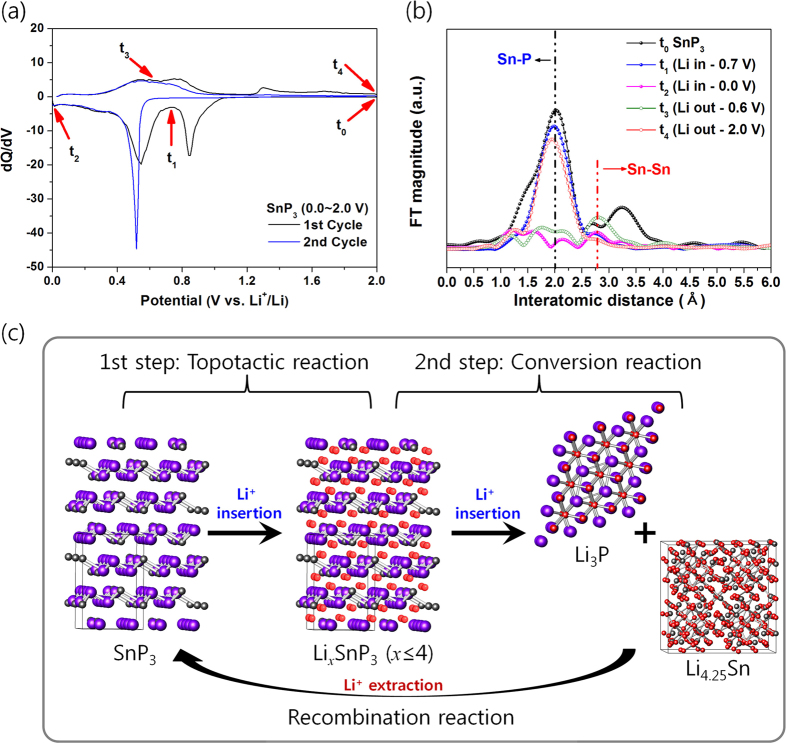
Reaction mechanism of layer-structured SnP_3_. (**a**) Differential capacity plot of the first cycle. (**b**) Sn K-edge EXAFS during the first cycle. (**c**) Schematic representation of the reaction mechanism of SnP_3_ with Li.

The DCP and cyclic voltammetry (CV) shows two large and broad peaks during both the discharging and charging reactions (Figs 2a and S2 in Supporting Information). The DCP and CV demonstrated that the electrode had two reactions during Li insertion/extraction, respectively. To evaluate the electrochemical Li reaction in the SnP_3_ electrode, *ex situ* XRD analyses were carried out at selected potentials (Fig. S3 in Supporting Information), as refered in the DCP results. However, no other peaks were detected in the *ex situ* XRD, with the exception of the full discharged state at 0.0 V. Therefore, to obtain the local structural variations in the SnP_3_ electrode, Sn K-edge EXAFS analyses (Fig. 2b) were corrected. The main EXAFS peak in the SnP_3_ spectra was associated with the Sn–P (2.0 Å) bond. In the discharged state at 0.7 V (t_1_ in Fig. 2b), the main Sn–P (2.0 Å) bond peak of SnP_3_ did not change. The corresponding capacity at 0.7 V was 1100 mA h cm^−3^, or 573 mA h g^−1^ as confirmed in Fig. 1c. Considered the capacity contributed to a SEI layer formation reaction was ca. 60 mA h g^−1^, nearly 4 moles of Li reacted per mole of SnP_3_, meaning SnP_3_ underwent a topotactic transition reaction as far as Li_*x*_SnP_3_ (*x* ≤ 4). When the electrode was in the fully discharged state at 0.0 V, *ex situ* XRD showed only the Li_3_P phase (Fig. S3 in Supporting Information). Additionally, the EXAFS peak relating to the Sn–P bond disappeared and a Li-interacted Sn–Sn peak appeared due to the formation of the Li_4.25_Sn alloy phase (t_2_ in Fig. 2b)^6^,^7^,^25^. These results show that SnP_3_ was fully converted into the Li_4.25_Sn and Li_3_P phases when Li was fully inserted. On the other hand, in the charged state at 0.6 V (t_3_ in Fig. 2b), only the Sn-Sn bond relating to metallic Sn was seen, demonstrating that Li_4.25_Sn transformed into Sn. In the fully charged state at 2.0 V (t_4_ in Fig. 2b), the peak indicating the presence of the Sn–P bond of SnP_3_ reappeared in the EXAFS spectra, which definitively demonstrates that SnP_3_ recombined after full Li extraction. Based on these results, the Li insertion/extraction mechanism during the first cycle of the SnP_3_ electrode can be summarized as follows:

During discharging:


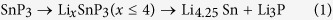


During charging:


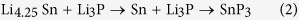


On the basis of this reaction mechanism, the SnP_3_ electrode can be seen to undergo topotactic and conversion reactions during discharging, and a recombination reaction during charging, respectively, which is schematically represented in Fig. 2c. The poor capacity retention of the SnP_3_ electrode confirmed by the voltage profile originated from the huge volume variation caused by the formation of conversed phases (Li_4.25_Sn and Li_3_P) and recombined phase (SnP_3_) during repeated Li insertion and extraction reactions, respectively.

**Figure 3:**
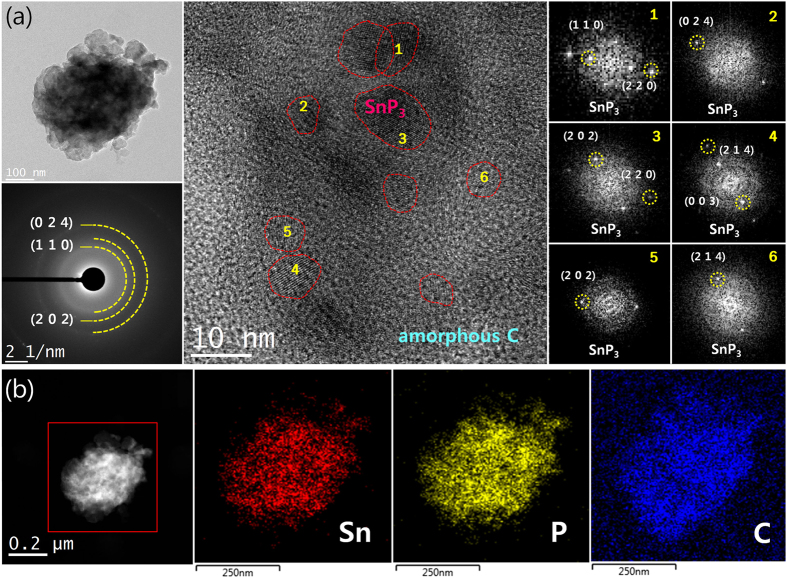
HRTEM images of the SnP_3_/C nanocomposite. (**a**) Bright-field TEM and HRTEM images with corresponding FT patterns. (**b**) EDS mapping images.

Recently, numerous researchers have reported that the preparation of nanostructured composites can alleviate the detrimental effects caused by large volume expansions of Li-alloy based materials^2^,^9^,^10^,^35^,^36^,^37^,^38^. Therefore, to improve the electrochemical performance of the SnP_3_ electrode, we produced a SnP_3_/C composite using an additional HEBM technique with amorphous carbon (Super P). According to the XRD pattern (Fig. S4 in Supporting Information), the SnP_3_/C composite was produced without any impurities. Figure 3 shows the bright-field transmission electron microscopy (TEM) and high-resolution TEM (HRTEM) images combined with selected area electron diffraction (SAED) and Fourier transform (FT) patterns of the SnP_3_/C nanocomposite. The HRTEM image and corresponding SAED and FT patterns show that approximately 5–10 nm-sized nanocrystalline SnP_3_ phases were embedded in amorphous carbon matrices. The energy-dispersive spectroscopy (EDS) elemental mapping image demonstrated that the SnP_3_ nanocrystallites were uniformly dispersed within the amorphous carbon matrices.

**Figure 4:**
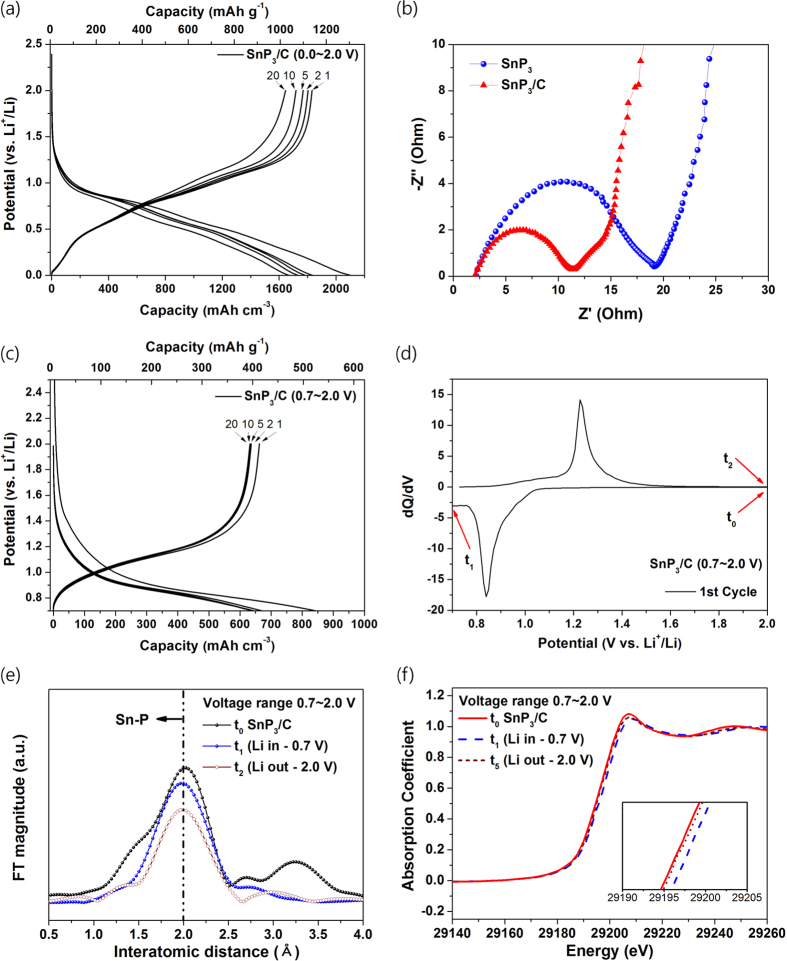
Electrochemical performances and reaction mechanism of SnP_3_/C nanocomposite electrode. (**a**) Electrochemical voltage profile of SnP_3_/C nanocomposite electrode at a current rate of 100 mA g^−1^ (voltage range: 0.0–2.0 V). (**b**) Comparison of electrochemical impedance results for SnP_3_ and SnP_3_/C electrode. (**c**) Voltage profile within the topotactic reaction region (voltage range: 0.7–2.0 V). (**d**) Differential capacity plot of the first cycle during the topotactic reaction. (**e**) Sn K-edge EXAFS results during the topotactic reaction. (**f**) Sn K-edge XANES results during the topotactic reaction.

Figure 4a shows the voltage profile of the SnP_3_/C nanocomposite electrode (voltage range: 0.0–2.0 V). The discharge/charge capacity of the electrode was 2103/1831 mA h cm^−3^ (1306/1137 mA h g^−1^), and it exhibited an excellent initial coulombic efficiency of 87.1%. Considered the capacity contributed to contents of ball-milled amorphous carbon (40 wt%) (Fig. S5 in Supporting Information), the SnP_3_ nanocrystallites within the composite underwent a highly reversible reaction, originating from the enhanced electrical conductivity provided by the preparation of the nanostructured SnP_3_/C composite. The enhanced electrical conductivity was confirmed by electrochemical impedance spectroscopy (EIS) analyses, as shown in Fig. 4b. The charge-transfer impedance of the SnP_3_/C nanocomposite electrode was much lower than that of the SnP_3_ electrode, indicating an even lower electrochemical reaction resistance in the nanostructured SnP_3_/C composite electrode, probably due to the uniform distribution of the SnP_3_ nanocrystallites within the conducting amorphous carbon matrices. Although the SnP_3_/C nanocomposite electrode showed greatly enhanced electrochemical performance compared with the SnP_3_ electrode, its capacity was reduced to 1645 mA h cm^−3^ after the 20th cycling.

The SnP_3_/C nanocomposite electrode was tested in the voltage range of 0.7–2.0 V and at a current density of 100 mA g^−1^ (Fig. 4c), in order to access the topotactic reaction between the SnP_3_ and Li_*x*_SnP_3_ (*x* ≤ 4). The first discharge/charge capacity was 840/663 mA h cm^−3^, with a high initial coulombic efficiency of approximately 78.9%. The contributed irreversible capacity of the ball milled amorphous C (40 wt%) in the potential range of 0.7–2.0 V was ca. 64 mA h g^−1^ (inset in Fig. S5a in Supporting Information). The electrode also showed excellent capacity retention, maintaining 95.5% of the initial charge capacity after the 20th cycling. The DCP of SnP_3_/C nanocomposite electrode in the potential range 0.7–2.0 V showed a large peak during both the discharging and charging reactions (Fig. 4d). To confirm the presence of the topotactic reaction, Sn K-edge EXAFS and XANES of the SnP_3_/C composite electrode were investigated within the voltage range of 0.7–2.0 V (Fig. 4e,f). In the discharged state at 0.7 V (t_1_ in Fig. 4e) and the charged state at 2.0 V (t_2_ in Fig. 4e), the main Sn–P (2.0 Å) bond peaks of SnP_3_ did not change. Additionally, the XANES spectra of the SnP_3_ electrode shifted to a slightly higher energy state at 0.7 V (t_1_ in Fig. 4f) and then returned to the energy state of SnP_3_ at 2.0 V (t_2_ in Fig. 4f), demonstrating that the valence state of Sn varied with the valence state of P. Therefore, the Li redox processes most likely occurred on P anion sites, with no structural variations, meaning the following topotactic reaction was firmly demonstrated:


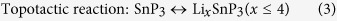


**Figure 5:**
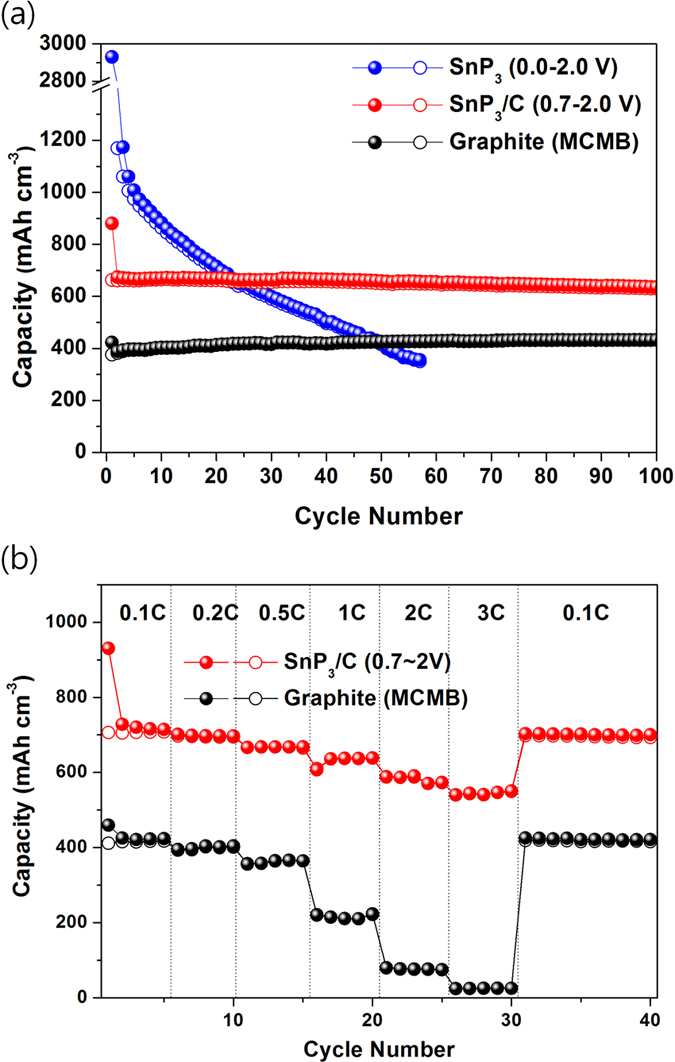
Comparison of electrochemical performances of SnP_3_, SnP_3_/C, and MCMB-graphite electrodes. (**a**) Cycling performances of SnP_3_ (voltage range: 0.0–2.0 V), SnP_3_/C (voltage range: 0.7–2.0 V), and MCMB-graphite (voltage range: 0.0–2.0 V) electrodes at a cycling rate of 100 mA g^−1^. (**b**) C-rate performances for the SnP_3_/C (voltage range: 0.7–2.0 V) and MCMB electrodes at various C rates.

Figure 5a shows the capacity versus the cycle number of SnP_3_ (voltage: 0.0–2.0 V), SnP_3_/C (voltage: 0.7–2.0 V), and graphite (mesocarbon microbead, MCMB) electrodes over 100 cycles. The SnP_3_ electrode exhibited poor cycling performance due to its low conductivity and large volume change during repeated Li reactions. However, when the SnP_3_/C nanocomposite electrode was tested within the topotactic reaction range (voltage: 0.7–2.0 V), it exhibited excellent cycling behavior having a high capacity of 636 mA h cm^−3^ over 100 cycles, which were higher capacity than that of the graphite electrode. The stable cycling behavior was attributed to the topotactic reaction between the SnP_3_ and Li_*x*_SnP_3_ (*x* ≤ 4) phases. The C-rate performances of the SnP_3_/C electrode and graphite were also tested. Figure 5b compares the C-rate performances of the SnP_3_/C nanocomposite (voltage: 0.7–2.0 V) and graphite electrodes (voltage: 0–2.0 V) as a function of the C rate, where C is defined as the full charge capacity after 1 h (SnP_3_/C: 700 mA h cm^−3^ and graphite: 400 mA h cm^−3^). The SnP_3_/C nanocomposite electrode exhibited much faster C-rate performance than the graphite electrode. At a cycling rate of 3C, the SnP_3_/C nanocomposite electrode showed a high charge capacity of ca. 550 mA h cm^−3^, which corresponded to ca. 78% of the charge capacity at 0.1C. The fast C-rate performance of the SnP_3_/C nanocomposite was ascribed to the short Li diffusion paths by preparation of extremely small SnP_3_ nanocrystallites (ca. 5–10 nm) within the conducting amorphous carbon matrices and enhanced electrical conductivity of the composite as confirmed in the EIS results.

## Conclusions

In summary, we synthesized layer structured SnP_3_ and demonstrated its reaction mechanism during Li insertion/extraction using various analytical techniques including *ex situ* XRD, EXAFS, and XANES analyses. During Li insertion, the SnP_3_ electrode showed sequential topotactic and conversion reactions, while a recombination reaction occurred after full Li extraction. When a nanosctructured SnP_3_/C composite electrode was tested within the topotactic reaction region (0.7–2.0 V), it showed a high volumetric capacity (1st charge capacity: 663 mA h cm^−3^), good capacity retention (636 mA h cm^−3^ over 100 cycles), and a high C-rate performance (550 mA h cm^−3^ at 3C). Based on these results, SnP_3_ and its composite materials were found to have interesting Li reaction mechanisms, and should be considered as alternative anode materials for LIBs.

## Methods

### Sample Preparation

SnP_3_ was synthesized by the following solid-state synthesis route: stoichiometric amounts of Sn (DAEJUNG, average size: ca. 45 μm, >99%) and P (Kojundo, average size: ca. 75 μm, >99%) powders were placed in an 80 cm^3^ hardened-steel vial along with stainless-steel balls (diameter: 3/8 in. and 3/16 in.) to give a ball-to-powder ratio of 20:1 by weight. This vial was assembled in an Ar-filled glove box, in which HEBM (Spex-8000) was conducted under an argon atmosphere for 12 h. For preparation of the nanostructured SnP_3_/C composite, the HEBM process for additional 2 h was carried out using the mixtures of the synthesized SnP_3_ powder and amorphous carbon (Super P, Timcal) as the raw materials. On the basis of the electrochemical performances of the nanostructured SnP_3_/C composite electrodes, optimal amounts of SnP_3_ and C to be 60% and 40% by weight, respectively, was revealed.

### Material characterization

The SnP_3_ and its composite (SnP_3_/C) samples were confirmed using XRD (DMAX2500-PC, Rigaku), HRTEM (FEI F20, operating at 200 kV), and EDS attached to the HRTEM. In addition, to observe the structure and phase changes occurring in the active SnP_3_ electrodes during Li insertion/extraction, *ex situ* XRD, XANES, and EXAFS analyses were performed. To avoid the air exposure of the electrodes, they were laminated using polyimide tape (Kapton) in an Ar-filled glove box. The Sn K-edge XANES and EXAFS spectra for the SnP_3_ and SnP_3_/C composite electrodes were recorded at the Pohang Light Source (PLS, 7D -XAFS beamline in a storage ring of 3.0 GeV) in Republic of Korea.

### Electrochemical measurement

For the electrochemical evaluation of the SnP_3_ and SnP_3_/C composite samples, electrodes were prepared by coating slurries onto Cu foil substrates. The slurries were consisted of the active material powder (80 wt%), conducting carbon black agent (Denka, 10 wt%), and polyvinylidene fluoride (PVDF, 10 wt%) binder dissolved in N-methyl-2-pyrrolidone (NMP) solvent. Coated slurries of each mixture were dried in vacuum at 120 °C for 3 h. The each electrodes were then pressed and punched. In an Ar-filled glove box, coin-type electrochemical cells were assembled using Li foil as the counter and reference electrodes, separator (Celgard 2400), and electrolyte (1 M LiPF_6_ in ethylene carbonate/diethyl carbonate (1:1 by volume, Panax STARLYTE)). With the exception of the C-rate tests, all electrochemical cells were examined galvanostatically at a current density of 100 mA g^−1^ between 0.0 and 2.0 V (vs. Li^+^/Li) using an automated battery cycling tester (Series 4000, Maccor), in which Li was inserted into and extracted from the working electrode on discharging and charging, respectively. The gravimetric capacity was calculated from the weight of the active materials, and the volumetric capacity was calculated by multiplying the gravimetric capacity by the tap density. The tap density results (MCMB-graphite: 1.27 g cm^−3^, SnP_3_: 1.92 g cm^−3^, SnP_3_/C composite: 1.61 g cm^−3^) were determined using a powder tap density analyzer (BT-301, Bettersize). CV results of the electrodes were tested using a potentiostat (SP-240, Bio-logic) in the voltage range of 0.0–2.0 V at a scanning rate of 0.15 mV s^−1^. EIS was conducted using an impedance analyzer (ZIVE-MP2A, WonATech), and potentiostatic impedance patterns were recorded over the frequency range of 10^5^ Hz to 10^−2^ Hz with an amplitude of 5 mV.

## Additional Information

**How to cite this article**: Park, J.-W. and Park, C.-M. Electrochemical Li Topotactic Reaction in Layered SnP_3_ for Superior Li-Ion Batteries. *Sci. Rep.* **6**, 35980; doi: 10.1038/srep35980 (2016).

## Supplementary Material

Supplementary Information
