# Supplementary material for: Electrochemical Li Topotactic Reaction in Layered SnP3 for Superior Li-Ion Batteries
Source: Sci Rep. 2016 Oct 24;6:35980. doi: 10.1038/srep35980 (PMC5075908; doi:10.1038/srep35980)
Supplement: Supplementary Information [file srep35980-s1.pdf]

Supplementary Information

Electrochemical Li Topotactic Reaction in Layered SnP_3 for Superior Li-Ion Batteries

Jae-Wan Park[a] *and Cheol-Min Park*[*a]

[a]School of Materials Science and Engineering, Kumoh National Institute of Technology, 61 Daehak-ro, Gumi, Gyeongbuk 39177, Republic of Korea

[*]Cheol-Min Park. Tel.: +82-54-478-7746; Fax: +82-54-478-7769

E-mail: cmpark@kumoh.ac.kr

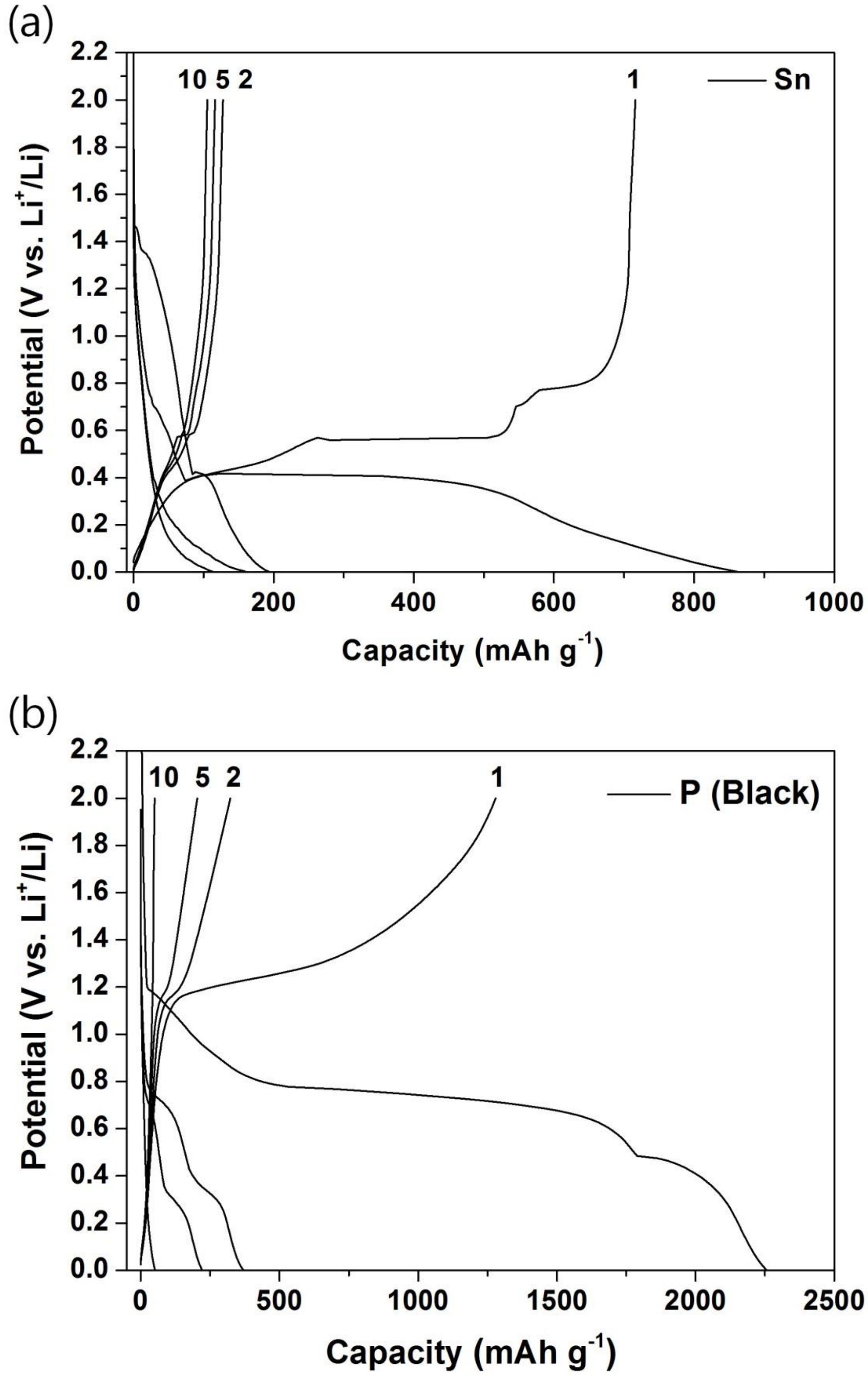

Figure S1. Electrochemical voltage profiles of Sn and P (black) electrodes at a current rate of 100 mA g^{-1} (voltage range: 0.0–2.0 V).

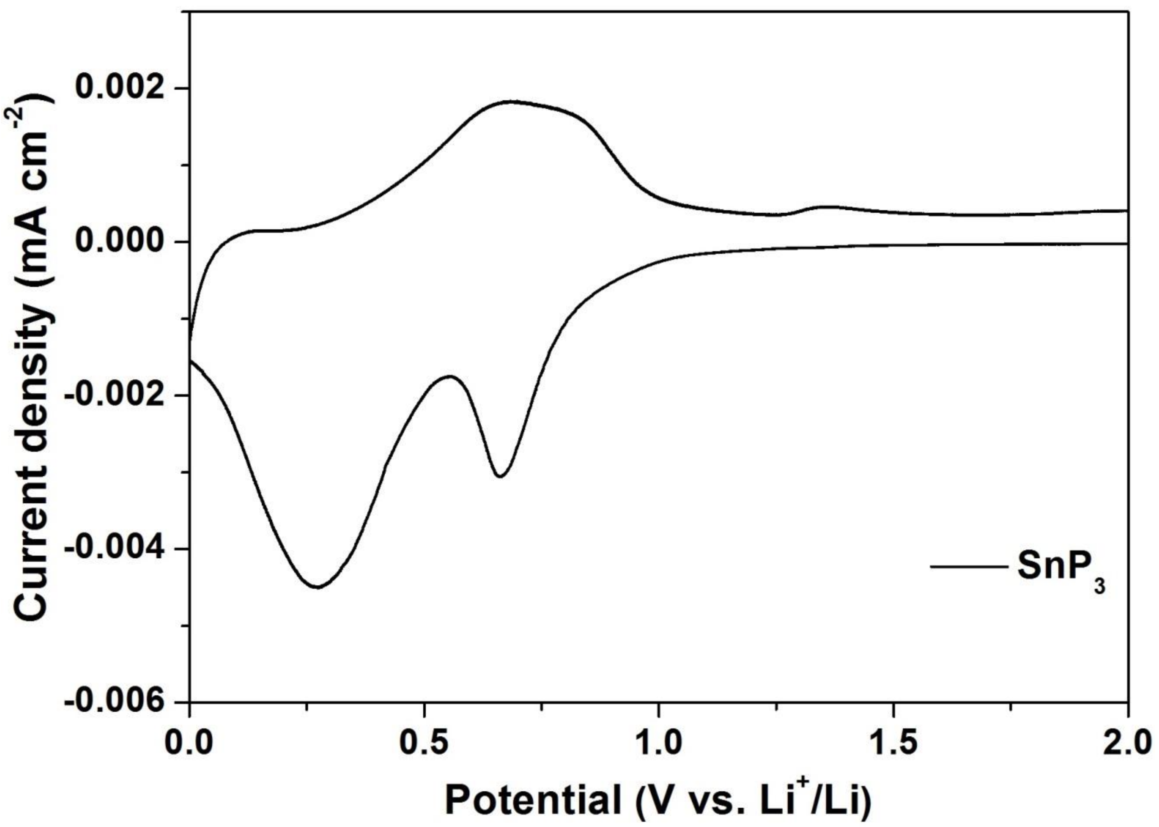

Figure S2. Cyclic voltammogram result for the first cycle of the SnP_3 electrode.

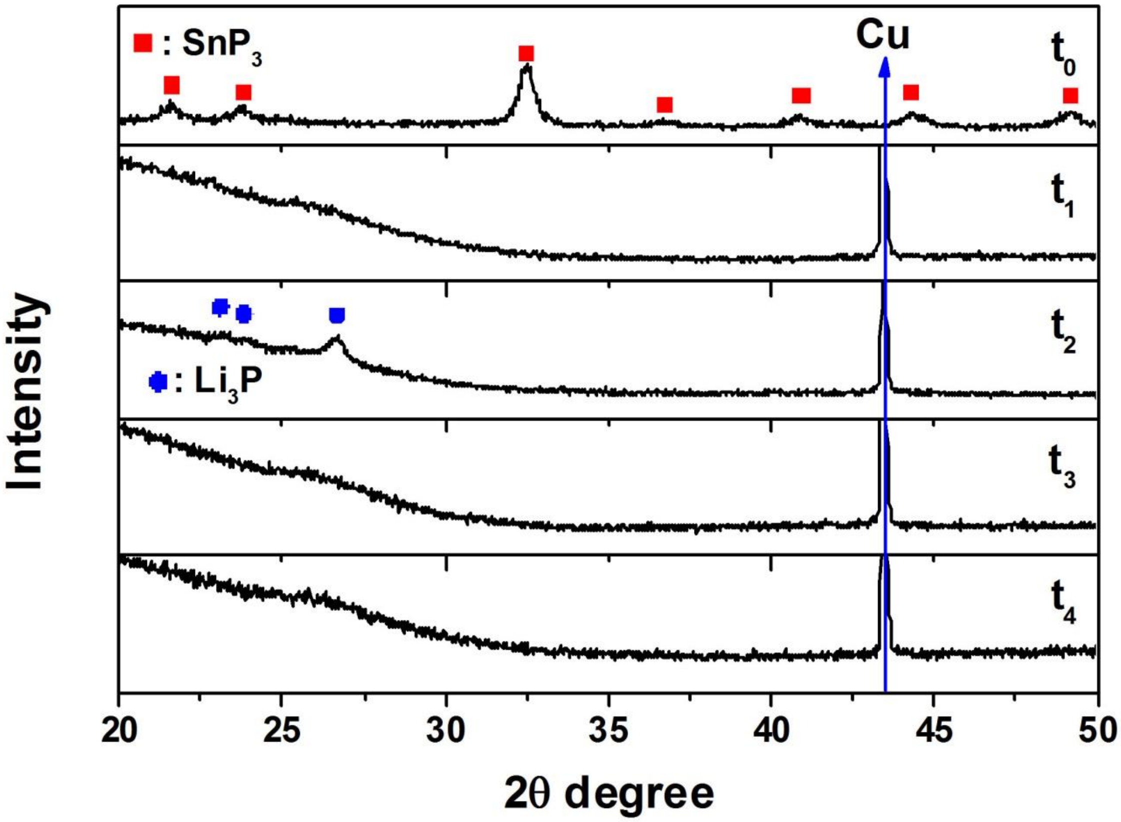

Figure S3. Ex situ XRD results of the SnP_3 electrode during the first cycle (t_x corresponds to the potentials indicated in Figure 2a).

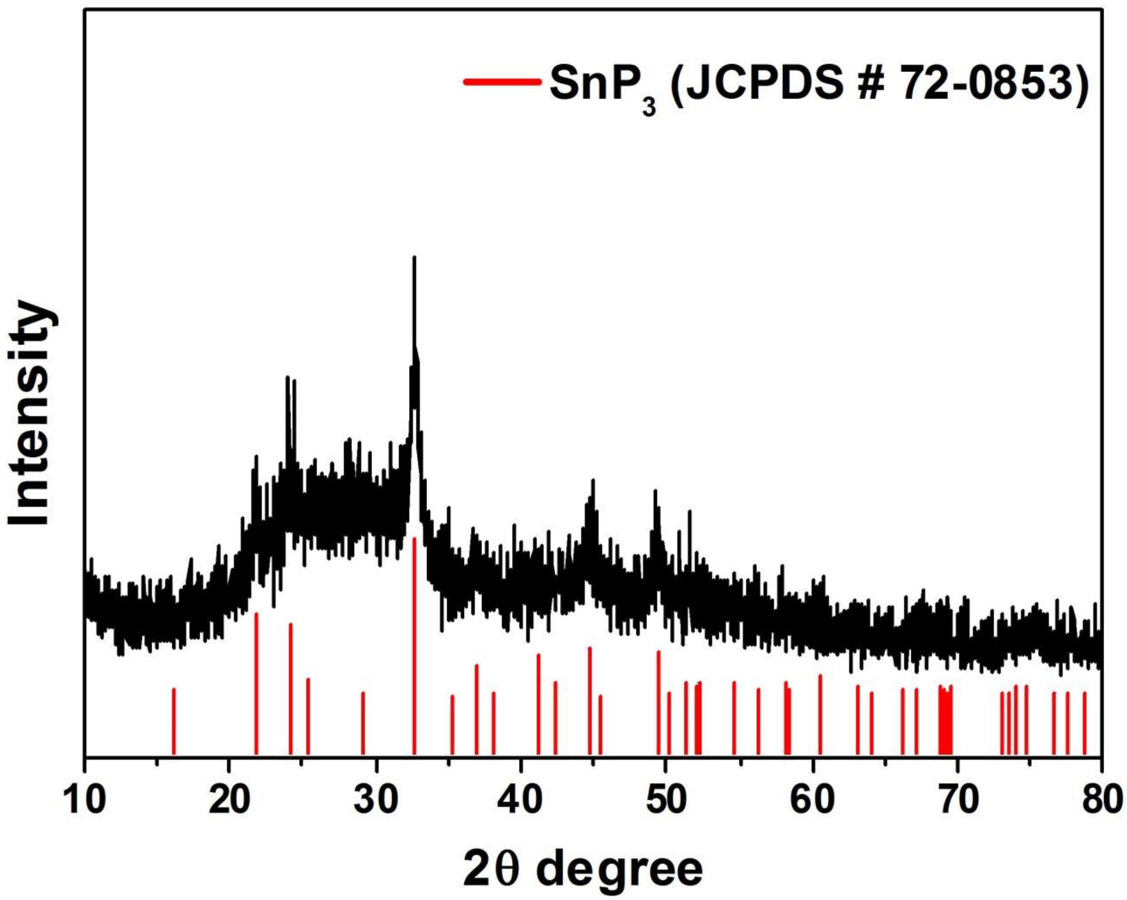

Figure S4. XRD pattern of the SnP_3/C nanocomposite prepared by the HEBM process.

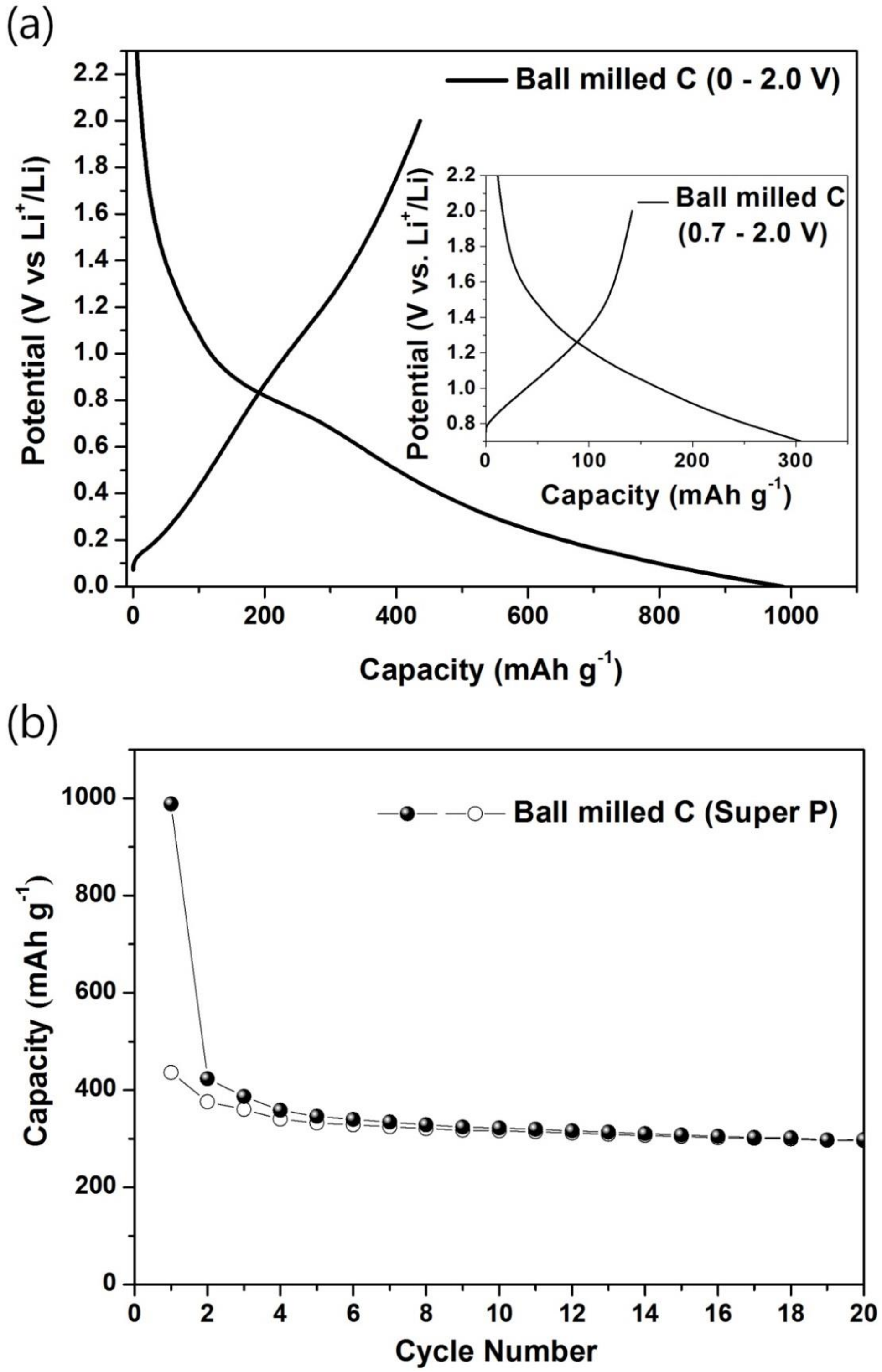

Figure S5. Electrochemical performances of ball milled C (Super P). (a) Voltage profile for ball milled C in the voltage range of 0–2.0 V and at a current density of 100 mA g^{-1} (Inset: voltage profile for ball milled C in the voltage range of 0.7–2.0 V). (b) Cycling performance of ball milled C (Super P) at a cycling rate of 100 mA g^{-1}.
